# Extended-pour and conventional alginates: effect of storage time on dimensional accuracy and maintenance of details

**DOI:** 10.1590/2177-6709.26.3.e2119251.oar

**Published:** 2021-06-30

**Authors:** Sandro Basso BITENCOURT, Isabela Araguê CATANOZE, Emily Vivianne Freitas da SILVA, Karina Helga Leal TURCIO, Daniela Micheline dos SANTOS, Daniela Atili BRANDINI, Marcelo Coelho GOIATO, Aimée Maria GUIOTTI

**Affiliations:** 1 Universidade Estadual Paulista, Faculdade de Odontologia de Araçatuba, Departamento de Materiais Odontológicos e Prótese (São Paulo/SP, Brazil).; 2 Universidade Estadual Paulista, Faculdade de Odontologia de Araçatuba, Departamento de Cirurgia e Clínica Integrada (São Paulo/SP, Brazil).

**Keywords:** Dimensional measurement accuracy, Alginates, Dental impression materials

## Abstract

**Objective:**

This study aimed to evaluate the dimensional stability and maintenance of details of conventional and high stability alginates up to 5-day storage.

**Methods:**

Two types of alginates were selected (n=10) for this study, conventional (Hydrogum) and high stability alginates (Hydrogum 5), which were produced with the aid of a cylindrical metal block and a ring-shaped metal mold (Specifications 18, 19, and 25, ANSI/ADA). Ten images were obtained from the molds for the dimensional stability test, which were taken immediately after their production and at each different storage periods (15 min, 24 h, 48 h, 72 h, 96 h, and 120 h) by a digital camera. The specimens were kept hermetically sealed in plastic bags (23°C) and then used to obtain 140 (n=70) dental stone models, used in the detail reproduction test, in which the angular accuracy of three grooves (20 µm, 50 µm, and 75 µm) was observed at each period. The details reproduction accuracy was classified using a predetermined score classification. Measurements of dimensional changes were made in the Corel DRAW X6 program. The data were submitted to the Student’s t-test (α?#8197;= 0.05).

**Results:**

A statistically significant difference concerning the size of the matrix was observed after 24h for both alginates, and a statistically significant negative linear dimensional change (contraction) was verified after 24 h of storage (1.52% for the high stability alginate, and 1.32% for the conventional alginate). The high stability alginate kept the full details for 72 hours, while the conventional alginate, for 24 h. Both alginates reproduced the 75 µm groove at all storage periods.

**Conclusion:**

Impressions made with both alginates presented satisfactory clinical results when the alginates were immediately poured.

## RESUMO

**Objetivo:**

O presente estudo teve como objetivo avaliar a estabilidade dimensional e manutenção de detalhes de alginatos convencionais e de alta estabilidade por até 5 dias de armazenamento.

**Métodos:**

Para esse estudo, foram selecionados dois tipos de alginatos: convencional (Hydrogum) (n = 10) e de alta estabilidade (Hydrogum 5) (n = 10), sendo produzidos com o auxílio de um bloco metálico cilíndrico e um molde metálico em forma de anel (especificações 18, 19 e 25, ANSI/ADA). Para o teste de estabilidade dimensional, dez imagens foram obtidas imediatamente e para cada tempo de armazenamento (15 min, 24, 48, 72, 96 e 120 horas), realizadas por câmera digital. As amostras foram mantidas hermeticamente fechadas em sacos plásticos (23°C). Para o teste de reprodução de detalhes, as amostras foram utilizadas para obter 140 (n = 70 por grupo) modelos de gesso, sendo observada a precisão angular de três sulcos (20 µm, 50 µm e 75 µm) para cada período. A precisão da reprodução de detalhes foi classificada usando uma classificação de pontuação predeterminada. As medições das mudanças dimensionais foram feitas no programa Corel DRAW X6. Os dados foram submetidos ao teste *t* de Student (α = 0,05).

**Resultados:**

Foi observada diferença estatística em relação à matriz após 24h para ambos os alginatos. Uma mudança dimensional linear negativa estatisticamente significativa (contração) foi verificada após 24 h de armazenamento (1,52% para alginato de alta estabilidade; 1,32% para alginato convencional). O alginato de alta estabilidade manteve os detalhes completos por até 72 horas, enquanto o alginato convencional, por 24 horas. Os alginatos reproduziram o sulco de 75 µm para todos os períodos.

**Conclusão:**

As impressões feitas com ambos os alginatos devem ser imediatamente vazadas para se ter resultados clínicos satisfatórios.

## INTRODUCTION

Optimal dental impression material should have dimensional stable performance over time and allow the pouring of the dental stone according to the convenience of the operator.^1^ Hydrocolloids and synthetic elastomeric polymers are the most frequently used materials to obtain dental impressions. When higher precision is required, some nonaqueous elastomeric impression materials can be used, like polysulfides, polyethers, and condensation or addition silicones.^2^ However, alginate is one of the most used impression materials, especially due to its satisfactory technical properties and low cost.

Their details reproduction allows creating stone cast models with good details of accuracy when correctly used. However, it is a very sensitive material, requiring a strict protocol of use. Certainly, one of the main causes of clinical failure when using alginate is its tendency to experience syneresis and imbibition, often as a result of mold storage issues, long time between molding and casting, and insufficient disinfection procedures.^3^ ^-^ ^6^ Alginate has poor detail reproduction and dimensional instability when stored for long periods.^5^ ^,^ ^6^ Therefore, its immediate pouring or right after disinfection is recommended. New products have been launched in the dental market to overcome some of these disadvantages inherent to the material composition, claiming to be dimensionally stable for up to 5 days (extended-pour alginates), being known as new generation of alginates due to their high stability.^3^ ^,^ ^7^ ^-^ ^10^

Dimensional stability is one of the most important properties of impression materials to obtain accurate dental stone models.^11^ The main limitation of alginate is its dimensional change after being removed from the mouth and stored for periods over 15 min.^12^ Thus, the analysis of dimensional stability and maintenance of details of these new high stability products is required, since the immediate pouring of the impressions may not always be possible, especially if the impression needs to be sent to a dental laboratory.^7^ Although this material is generally used to obtain diagnostic casts, it is frequently used during the manufacturing of the partially removable dental prostheses.^6^ ^,^ ^11^ Therefore, the knowledge about the dimensional behavior of impression materials is of utmost importance, and this study aimed to provide new information to the clinician about the different products available in the market, providing scientific support to the selection of the best materials.

This study aimed to evaluate the influence of 5-days storage on the dimensional stability and maintenance of details of molds obtained from two types of alginate (conventional and high stability). Two null hypotheses were tested, in which there was no statistically significant difference between both alginates, regarding their dimensional stability; and that the dimensional stability and maintenance of details would not be affected by the storage time.

## MATERIAL AND METHODS

**Figure 1:**
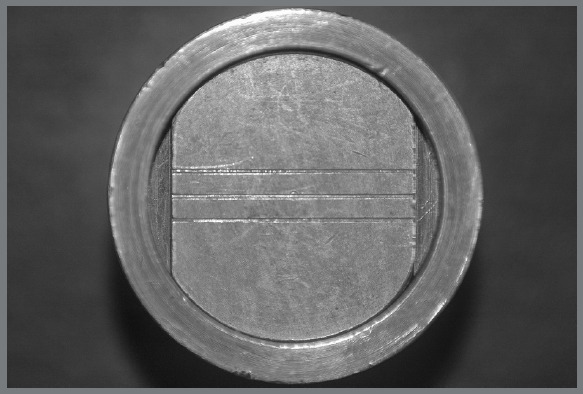
Metallic cylindrical matrix.

Two types of alginates were used to make the test specimens, the conventional and high stability alginates (Hydrogum^®^ and Hydrogum 5^®^, Zhermack Spa, Badia Polesine, Italy). The test specimens were prepared according to ANSI/ADA specifications#18, 19, and 25,^13^ ^-^ ^15^ using a cylindrical metal block and a ring-shaped metal mold (Fig. 1) in order to test their dimensional stability and maintenance of details. The ring-shaped metal mold was adapted to the upper part of the cylindrical metal block, to leave a space where the material was placed. 

The alginates were manipulated according to the instructions of the manufacturer at 23 ± 2°C and at relative humidity of 50 ± 10%. After manipulation, the material was placed in the mold using a spatula. After insertion, a polyethylene strip was laid, followed by a glass plate. A 1,500 g load was placed on the glass plate to extrude the excess of impression material, and kept under pressure. Subsequently, the material was immersed in distilled water at 35 ± 1°C and kept in an incubator for three min longer than the minimum time recommended by the manufacturer. After this time, the set was removed from the incubator, the mold separated from the metal matrix, and the specimens were carefully removed to avoid distortions.

### LINEAR DIMENSIONAL CHANGE TEST

**Figure 2:**
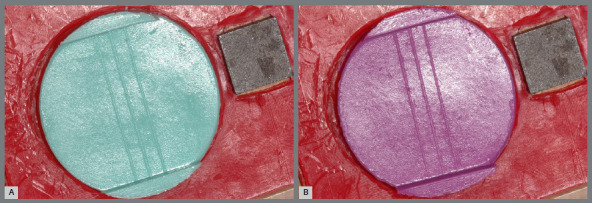
Specimens of conventional Hydrogum^®^ (A) and Hydrogum 5^®^ (B).

Twenty specimens (n=10) were made and, since the same test specimen was used for the different storage times, variables such as water/powder ratio, hand spatulation time, etc, were excluded. Photos were taken using a digital camera (Nikon D50) with a macro lens and a ring flash, mounted on the stand (Asahi Pentax), with the camera distance/object determined and maintained equal for all specimens. Along with the specimen, a 10 x 10 mm metal block was placed (a metallic reference standard) to determine the true magnitude of the picture, enabling to scale the values obtained in actual numbers (Fig. 2A and B). Each specimen (n=10) was photographed immediately after its removal from the matrix and at each storage period (15 min, 24 h, 48 h, 72 h, 96 h, and 120 h). 

**Figure 3:**
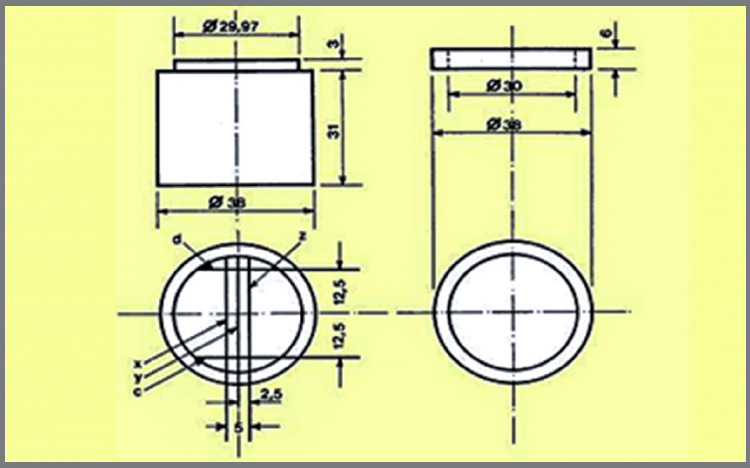
Schematic drawing of the matrix.

**Figure 4:**
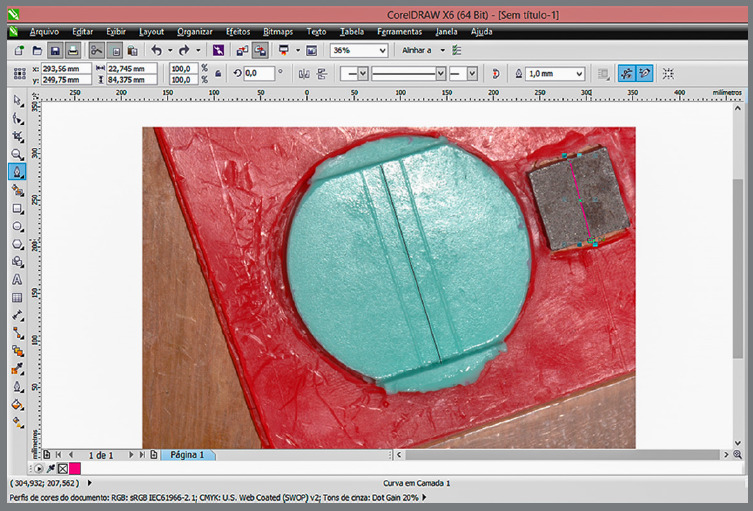
Image measurement in the Corel DRAW X6 program.

The specimens were stored according to the instructions of the manufacturer in hermetically sealed plastic bags at room temperature (23°C), placed at the laboratory bench during all the experimental periods. Ten images were obtained for each time of storage for each material, divided into seven groups. After the image capture, the specimen returned to the hermetically sealed plastic bag and the process was repeated for each storage time. The linear dimensional alteration was analyzed based on the distance between edges C’ and D’, measured in the pictures taken, using a computer program (Corel DRAW X6, Corel Corporation). The original distance between these edges was of 25 mm (Fig. 3, the original distance of the matrix). The measurement of each image was repeated three times to obtain an average value (Fig. 4). The percentage of dimensional change of the alginate molds tested was calculated using the formula expressed below: 

Dimensionalchange(%)=(B−A)/A×100

A = original distance of the block between edges C and D = 25 mm.

B = distance between edges C’ and D’ in the test specimens, after storage periods.

### MAINTENANCE OF DETAILS TEST

A total of 70 specimens for each alginate mold (n=10) were prepared and divided according to the storage period (immediately, 15 min, 24 h, 48 h, 72 h, 96 h, and 120 h) for the maintenance of details test. After each storage period, the molds were inserted in a frame and the pouring of the dental stone type IV (Durone, Dentsply, Petrópolis, Brazil) occurred under mechanical vibration after the proportioning and vacuum tooling, according to the manufacturer, which were separated 1 hour after the pouring.

The maintenance of details of the dental stone models was tested in a stereomicroscope (Olympus) under a low-angle illumination at 13× magnification. In the detail reproduction test, the angular accuracy of three grooves (x =50 ? 5 µm; y =20 ? 5 µm; z =75 ? 5 µm) molded in each sample was recorded, obtained after the following storage periods: 15 min, 24, 48, 72, 96 and 120 h. To classify the detail reproduction accuracy, the scores suggested by Goiato et al.^16^ were used, as follows: 0 - full reproduction of two of the three grooves; 1 - full reproduction of the three grooves, with inaccurate angles; 2 - full reproduction of the three grooves, with accurate angles.

### STATISTICAL ANALYSIS

All the photos were taken by a single examiner and under the same camera setup, to avoid discrepancies between the images. After capturing the images, they were transferred to the computer and analyzed using the Corel DRAW software. The measurements were repeated twice after a two-days interval, to examine the reproducibility of the method, by statistical analysis; and for intraclass evaluation. After performing two measurements every two days between them, the average result obtained was CCI = 0.90 (F = 17.91; *p* = 0.0001), indicating reproducibility of the method.

The data were submitted to statistical analysis using the Student’s t-test (paired samples), and the two alginates were compared using the Student’s t-test (SPSS 20.0, IBM), at the level of confidence of 95% (*p*< 0.05).

## RESULTS

### LINEAR DIMENSIONAL CHANGE RESULTS

The frequency distribution of each measurement was analyzed for the assumption of normality, which was confirmed for the quantitative data. Table 1 shows the mean values and standard deviations of the dimensional changes that occurred in the conventional and high stability alginates during all the storage period. The results showed that there was no significant difference regarding the C and D dimensions of the metal matrix (25 mm), immediately after its production and at the 15 min storage time (Table 1). Both alginates presented a negative linear dimensional change (contraction) statistically significant after 24 h of storage (1.52% for the high stability alginate and 1.32% for the conventional alginate). 

**Table 1:** Mean values (SD) of dimensional change of conventional and high stability alginates at different storage periods (mm and %).

Storage periods	Conventional		High stability	
Control (matrix)	25.00 (0)^Aa^	0	25.00 (0)^Aa^	0
Immediate	25.01 (0.23)^Aa^	+0.04	25.01 (0.18)^Aa^	+0.04
15 min	25.08 (0.31)^Aa^	+0.32	25.13 (0.37)^Aa^	+0.52
24 hours	24.67 (0.24)^Ba^	-1.32	24.62 (0.51)^Ba^	-1.52
48 hours	24.35 (0.39)^BCa^	-2.60	24.32 (0.4)^BCa^	-2.72
72 hours	24.34 (0.35)^Ca^	-2.64	24.54 (0.51)^BCa^	-1.84
96 hours	24.30 (0.4)^Ca^	-2.80	24.29 (0.22)^BCa^	-2.84
120 hours	24.41 (0.43)^BCa^	-2.36	24.35 (0.47)^Ca^	-2.60
General average	24.59 mm (-1.62%)	-1.62%	24.61 mm (-1.56%)	-1.56%

Means followed by the same capital letters in the column and the same lowercase letters in the row did not differ statistically from each other at the level of 95% reliability (p<0.05).

**Figure 5:**
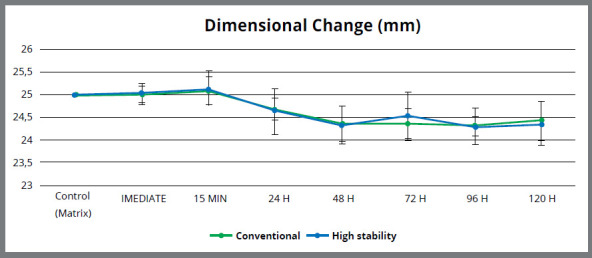
Line graph of the dimensional change ( mm ) in all the periods evaluated.

There was no statistically significant difference for the high stability alginate from 24 h to 96 h of storage time. After the maximum period of time recommended by the manufacturer for the storage of the material (120 h), the high stability alginate suffered an average contraction of 1.56%. Regarding the conventional alginate, there were no statistically significant difference between the storage periods of 24 h and 48 h and between 48 h to 120 h. Further, there was no statistically significant difference between the alginates, regardless of storage periods (Fig. 5).

### MAINTENANCE OF DETAILS

**Figure 6:**
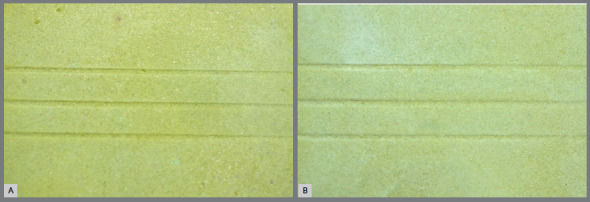
Stone cast models with a reproduction of the 75 µm groove: A) high stability and B) conventional alginate.

Tables 2 and 3, and Fig. 6 (A and B) show the results of the maintenance of details test for the two alginates, depending on the storage time, according to the classification of Goiato et al.^16^ In this scale, score 0 is assigned to full reproduction of two of the three grooves; score 1, to full reproduction of the three grooves, without accurate angles; and score 2, to full reproduction of the three grooves, with accurate angles.

**Table 2:** Scores of maintenance of details for casts obtained from high stability alginates, as a function of the storage time.

SCORE CLASSIFICATION						
Stone cast models	15 min	24 hours	48 hours	72 hours	96 hours	120 hours
Specimen 1	Score 2	Score 2	Score 2	Score 2	Score 2	Score 2
Specimen 2	Score 2	Score 2	Score 2	Score 2	Score 2	Score 2
Specimen 3	Score 2	Score 2	Score 2	Score 2	Score 2	Score 2
Specimen 4	Score 2	Score 2	Score 2	Score 2	Score 1	Score 2
Specimen 5	Score 2	Score 2	Score 2	Score 2	Score 1	Score 2
Specimen 6	Score 2	Score 2	Score 2	Score 2	Score 2	Score 2
Specimen 7	Score 2	Score 2	Score 2	Score 2	Score 2	Score 1
Specimen 8	Score 2	Score 2	Score 2	Score 2	Score 2	Score 2
Specimen 9	Score 2	Score 2	Score 2	Score 2	Score 2	Score 1
Specimen 10	Score 2	Score 2	Score 2	Score 2	Score 2	Score 2
Score 2 reproduction	100%	100%	100%	100%	80%	80%

**Table 3:** Scores of maintenance of details for casts obtained from conventional alginates, as a function of the storage time.

SCORE CLASSIFICATION						
Stone cast models	15 min	24 hours	48 hours	72 hours	96 hours	120 hours
Specimen 1	Score 2	Score 2	Score 2	Score 2	Score 2	Score 1
Specimen 2	Score 2	Score 2	Score 2	Score 2	Score 2	Score 1
Specimen 3	Score 2	Score 2	Score 2	Score 2	Score 1	Score 1
Specimen 4	Score 2	Score 2	Score 2	Score 2	Score 1	Score 1
Specimen 5	Score 2	Score 2	Score 2	Score 1	Score 2	Score 1
Specimen 6	Score 2	Score 2	Score 2	Score 1	Score 1	Score 1
Specimen 7	Score 2	Score 2	Score 2	Score 1	Score 2	Score 1
Specimen 8	Score 2	Score 2	Score 1	Score 1	Score 1	Score 1
Specimen 9	Score 2	Score 2	Score 1	Score 1	Score 1	Score 1
Specimen 10	Score 2	Score 2	Score 2	Score 2	Score 2	Score 1
Score 2 reproduction	100%	100%	80%	50%	50%	0%

In the high stability alginate (Table 2), the maintenance of details test presented a score 2 (full reproduction of the three grooves, with accurate angles) for 100% of the specimens for the storage time of 15 min, 24 h, 48 h, and 72 h. The same score was obtained for 80% of samples for the groups subjected to storage periods of 96 h and 120 h.

**Figure 7:**
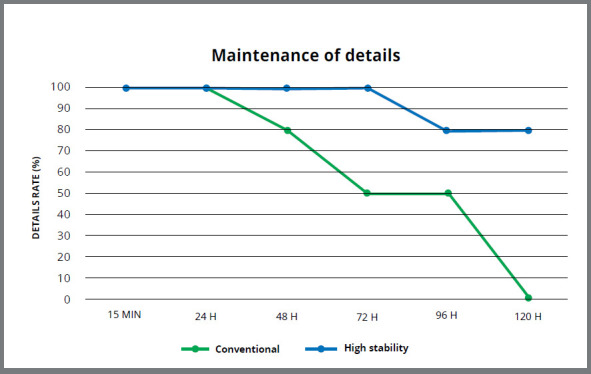
Line graph of the rate of maintenance of the details (%).

Regarding the conventional alginate (Table 3), the maintenance of details presented a score 2 (total reproduction of the three grooves, with accurate angles) in 100% of the samples for storage periods of 15 min to 24 h. The groups subjected to storage periods of 48 h, 72 h, 96 h, and 120 h, the same score was obtained in 80%, 50%, 50%, and 0% of the samples, respectively (Fig. 7). Following the ADA specification #18,^13^ both alginates reproduced the groove of 75 µm in width, fulfilling properly this requirement.

## DISCUSSION

Alginates are used currently, day after day at dental offices, especially due to their several advantages. They present low cost, well acceptability by the patient, easy manipulation and execution technique, as well the possibility of achieving a detailed impression all in a single step, being outstanding for primary prosthetic, orthodontic and design imprints. However, alginates present some disadvantages like lower accurate reproduction than elastomeric impression materials, and poor dimensional stability for complex cases.^17^

In this study, the linear dimensional change (% mean) for high stability alginate and conventional alginate at 24 h of storage was of -1.52% and -1.32%, respectively (Table 1). The values were statistically different from the immediate period after mold production and at 15 min of storage. These data revealed that the alginate molds, once removed from the mouth, experience some contraction associated with syneresis and evaporation.^11^ According to the manufacturer, if the gypsum can not be immediately poured, the impression should be stored in a bag sealed at room temperature (23°C), and delayed for up to 5 d after taking the impression. In this study, however, the molds of high stability alginate remained dimensionally stable only in the first 15 min. The same result was obtained with the conventional alginate.

In the methodology used in this study, the molds were stored in sealed bags without relative humidity, following the recommendations of the manufacturer. According to literature guidelines,^11^ when they need to be stored, this must be done in containers with a relative humidity of 100%. This factor might explain the unsatisfactory or less than expected results obtained.

The ADA specification #18^13^ does not stipulate a maximum clinically acceptable value for the dimensional changes of alginates.^3^ ^,^ ^9^ ^,^ ^18^ ^,^ ^19^ However, for the elastomeric materials, these values ​​are well defined in the literature and may not exceed 1%, according to the ANSI/ADA specification #19.^14^ The polysulfides present a shrinkage percentage in 24 h that vary from 0.4% to 0.45%; condensation silicones, from 0.38% to 0.6%; addition silicones, from 0.14% to 0.17%; and polyethers, from 0.19% to 0.24%.^11^ There are controversies regarding the dimensional change values considered clinically acceptable for alginates, ranging from 0.1% to 0.8%.6 According to Imbery et al.^3^ and Rohanian et al.,^9^ for a plaster model to be considered clinically acceptable, it should not present a discrepancy ​​higher than 75 µm from the originally intended size. The authors considered as a clinically acceptable value the amount of 0.5% dimensional change for alginates.

Although dentists do not use alginates to obtain impressions for fixed partial denture, *in vitro* and *in vivo* studies determined that the misfit of the prosthetic crown should not exceed the range of 25-80 µm, approximately.^11^ ^,^ ^20^ ^-^ ^22^ However, there is no consensus in the literature about the acceptable values for this maladjustment. According to the ADA specification #8,^23^ the marginal crown adaptation in the cementing procedure should be within the range of 25 µm, although many studies reported as clinically acceptable values less than or equal to 120 µm for the good performance of these crowns.^24^ ^,^ ^25^

In this study, for high stability alginate, after 24 h of storage, the mean linear dimensional change was 24.62 mm, with an inaccuracy of 0.38 mm (380 µm) concerning the value of the matrix (25 mm). The highest mean value of dimensional change in this material was observed in the group that remained stored for 96 h (24.29 mm), presenting an inaccuracy of 0.71 mm (710 µm). The high stability alginate showed a mean linear shrinkage of 1.56% (24.61 mm), which in numerical terms was equivalent to a discrepancy of 0.39 mm (390 µm), well above the acceptable for its use as a molding material for FPD.

Therefore, this study showed that alginate, regardless of its high dimensional stability, does not present enough accuracy to be used in impressions that require a high accuracy level, unless the dental stone is poured within 15 min. This fact is predictable given that this type of material is not designed to be stored for long periods. Most authors recommend to pour the dental stone immediately or at the most in 15 min,^13^ since the accuracy of the mold may easily change due to syneresis and imbibition. On the other hand, different results are found in the literature regarding the high stability alginates. In the study of Imbery et al.,^3^ the high stability alginate investigated (Cavex Color change) showed a dimensional change from 0.16% to -0.49% during the 5 days of storage. The authors concluded that when the high stability alginate is properly stored, it can produce accurate impressions for up to 5 days. However, they emphasized that the impressions obtained by these materials only had the purpose of making study stone cast models or manufacturing acrylic appliances. Rohanian et al.^9^ also found satisfactory results for the alginates Hydrogum 5 and Alginoplast, stating that the impressions of these materials can be stored for up to 120 h and 72 h, respectively, without significant dimensional changes. 

Differences in the results found in the literature may be related to the different methodologies employed (matrix types, conditions under which samples were stored, different trademarks, and so on). Todd et al.^10^ found unsatisfactory results, observing that the tested high stability alginates (Kromopan and Triphasix) showed significant dimensional changes after 24 h and 100 h of storage. These data corroborate the results of the present study, in which the high stability alginate experienced statistically significant dimensional change after 24 h of storage. The matrix used by Todd et al.^10^ was similar to that used in this study.

Regarding the maintenance of details, the analysis was made for the total continuity and sharpness of angles of three grooves molded in each sample (width of groove: x = 50 ? 5 µm; y = 20 ? 5 µm; z = 75 ? 5 µm). According to the specification # 18 ANSI/ADA,^13^ plaster models obtained from alginate should reproduce the groove of 75 µm in width. Thus, following the ADA recommendation, both alginates reproduced the groove of 75 µm in width, meeting this requirement satisfactorily. 

Therefore, the first null hypothesis of this study was confirmed, with no statistically significant difference being observed between the alginates. The second hypothesis was rejected, showing that the dimensional stability and the maintenance of reproduction details were affected by the storage time for both alginates. Few studies have investigated the dimensional accuracy of high stability alginates^3^ ^,^ ^9^ ^,^ ^10^ ^,^ ^26^ ^,^ ^27^ and, considering the controversial results found in the literature, alginate impressions should be stored for the shortest period of time. The dimensional stability of the molds of both alginates was affected by the storage time. However, both alginates reproduced the 75 µm groove at all storage periods. Regarding clinical application, the present results suggest that impressions made with both alginates would be most precise if poured within 15 minutes. Currently, the study of alternative impression materials, such as irreversible hydrocolloids, is increasing, aiming to overcome the issues associated with the water-based irreversible hydrocolloid impressions. Future studies testing the dimensional stability and the maintenance of reproduction details comparing different impression materials are encouraged.

## CONCLUSION

The findings of this study showed that impressions made with these materials should be immediately poured to have satisfactory clinical results, since dimensional alterations were found after 24 hours, even on high stability alginates.
